# Bioinformatic Evidence Reveals that Cell Cycle Correlated Genes Drive the Communication between Tumor Cells and the Tumor Microenvironment and Impact the Outcomes of Hepatocellular Carcinoma

**DOI:** 10.1155/2021/4092635

**Published:** 2021-10-26

**Authors:** Dongdong Chen, Zhijun Feng, Mingzhen Zhou, Zhijian Ren, Fan Zhang, Yumin Li

**Affiliations:** ^1^Lanzhou University Second Hospital, No. 82, Cuiyingmen, Chengguan District, Lanzhou, Gansu, China; ^2^Key Laboratory of Digestive System Tumors of Gansu Province, No. 82, Cuiyingmen, Chengguan District, Lanzhou, Gansu, China; ^3^Gansu Provincial Hospital, No. 204, Donggang West Road, Chengguan District, Lanzhou, Gansu, China; ^4^Southern University of Science and Technology Hospital, No. 6019, Xili Liuxian Avenue, Nanshan District, Shenzhen, Guangdong, China

## Abstract

Hepatocellular carcinoma (HCC) is an aggressive cancer type with poor prognosis; thus, there is especially necessary and urgent to screen potential prognostic biomarkers for early diagnosis and novel therapeutic targets. In this study, we downloaded target data sets from the GEO database, and obtained codifferentially expressed genes using the limma *R* package and identified key genes through the protein–protein interaction network and molecular modules, and performed GO and KEGG pathway analyses for key genes via the clusterProfiler package and further determined their correlations with clinicopathological features using the Oncomine database. Survival analysis was completed in the GEPIA and the Kaplan–Meier plotter database. Finally, correlations between key genes, cell types infiltrated in the tumor microenvironment (TME), and hypoxic signatures were explored based on the TIMER database. From the results, 11 key genes related to the cell cycle were determined, and high levels of these key genes' expression were focused on advanced and higher grade status HCC patients, as well as in samples of TP53 mutation and vascular invasion. Besides, the 11 key genes were significantly associated with poor prognosis of HCC and also were positively related to the infiltration level of MDSCs in the TME and the HIF1A and VEGFA of hypoxic signatures, but a negative correlation was found with endothelial cells (ECs) and hematopoietic stem cells. The result determined that 11 key genes (RRM2, NDC80, ECT2, CCNB1, ASPM, CDK1, PRC1, KIF20A, DTL, TOP2A, and PBK) could play a vital role in the pathogenesis of HCC, drive the communication between tumor cells and the TME, and act as probably promising diagnostic, therapeutic, and prognostic biomarkers in HCC patients.

## 1. Introduction

Hepatocellular carcinoma (HCC), an aggressive cancer type with poor prognosis, ranks fifth in cancer incidence worldwide and is the second most frequent cause of cancer-related mortality [1, 2]. In terms of pathophysiology, it is widely considered that HCC is an inflammation-driven disease [3]. Chronic inflammation, long-term injury, and regeneration processes perpetuate liver fibrosis and result in distortion of lobular architecture, nodular formation, and cirrhosis; the dysplastic cirrhotic nodules then continue to evolve and eventually develop into early-stage and advanced HCC [4, 5]. To reduce the risk of hepatocarcinogenesis, adequate monitoring of symptoms, follow-up and evaluation of disease status, and improved early diagnosis and targeted therapies are particularly important for patients with chronic liver disease who are at high risk of HCC. Unfortunately, few biomarkers have been incorporated for the evaluation and detection of the stage of progression from liver cirrhosis to liver cancer. In terms of treatment, liver transplantation still remains the only curative option for patients with cirrhosis and HCC, but it is limited to a selected group rather than all patients [6, 7]. While surgical resections can cure patients at early-stage of HCC [8], the reality is, however, that most patients are not suitable for potentially curative therapy due to the high burden of liver disease, extra-hepatic spread, poor background liver function related to cirrhosis, or the advanced stage at the time of diagnosis [9, 10]. Therefore, the identification of novel and effective markers for early warning for HCC and the exploration of new therapeutic targets for liver diseases are urgently needed.

From another perspective, serum alpha-fetoprotein (AFP) is a biomarker for HCC patients that have been widely used for several decades, but now, it has been found to possess limited sensitivity [11–13]. Therefore, new biomarkers for early diagnosis, prediction of recurrence, and assessment of overall survival (OS) are urgently needed. Tumor development is driven by complex patterns of genetic and epigenetic abnormalities [14], and HCC is no exception. Genetic alterations may be the potential “drivers” during the process of hepatocarcinogenesis. Clearly, knowing which genes are related to hepatic cirrhosis and disrupting the persistent progression of cirrhosis can be helpful in the inhibition of the progression of liver cirrhosis toward end-stage liver disease. However, these initiatives are still a significant clinical challenge. Therefore, it is necessary to explore the genetic changes and potential molecular mechanisms of the occurrence and development of hepatic cirrhosis to HCC. On one hand, it can help us to find more specific biomarkers for diagnosis and assessment of prognosis. On the other hand, it has a significant guiding role for us to better design individualized regimens, especially targeting therapies.

In this study, we selected data sets from the Gene Expression Omnibus (GEO) database [15] and firstly explored differentially expressed genes (DEGs) in tissues with hepatic cirrhosis; we then integrated the DEGs with the genes screened by comparing HCC and normal liver tissues, obtaining coexpressed DEGs. Furthermore, through the molecular modules of DEGs, we identified key genes that were critical to the development of cirrhosis and HCC. To further investigate the key genes in the diagnosis and prognosis of HCC, we explored their relationships with clinical pathological features of HCC patients using the Oncomine database [16, 17] and evaluated their effects on HCC prognosis. Finally, to clarify the key genes that drive communication between tumor cells and the tumor microenvironment (TME), we also assessed their associations with the TME, including immune and hypoxia microenvironments of HCC. This work helped us to clearly understand the genetic changes from hepatic cirrhosis toward HCC and provided new evidence for these genes to be used as reliable biomarkers for early diagnosis, prognosis assessment, recurrence monitoring, and therapeutic targets for patients with HCC.

## 2. Materials and Methods

### 2.1. Microarray Data

We retrieved candidate datasets in the GEO database (https://www.ncbi.nlm.nih.gov/geo/) with “liver” and “GPL570” as the key words. Furthermore, three datasets annotated by the “GPL570” platform were selected for their inclusion in this study. The GSE84044 [18] data set provides a characterization of the gene expression profile from patients with varying degrees of hepatitis B virus-related (HBV-related) liver fibrosis patients. GSE112790 [19] provides a comprehensive molecular characterization of liver cancer. GSE107170 [20] provides a gene expression profile of tissue specimens from livers with HBV-HCC and hepatitis C virus-related (HCV-related) HCC. We downloaded the gene expression matrix files of these target data sets, and data processing (extracting, standardizing, log2-transforming) was performed using *R* software (Version R 3.6.1, https://cran.r-project.org/) and related packages (http://www.bioconductor.org/).

### 2.2. Screening for DEGs

Differential analysis was performed using the limma package [21], between each of the following samples: samples with liver cirrhosis vs. samples with nonliver cirrhosis, samples with HCC (no specific history of liver disease) vs. samples with non-HCC, samples with HBV-related HCC vs. samples with HBV-infection but nontumor, and samples with HCV-related HCC vs. samples with HCV-infection but nontumor. A volcano map was plotted to assess the differential expression of all genes using the ggplot2 package (https://cran.r-project.org/web/packages/ggplot2/index.html). As a DEG, it was necessary to satisfy both statistical *P* < 0.05 and ∣log fold change (FC) | >1 [22]. The coexpressed DEGs were obtained and then visualized using the UpSetR package [23].

### 2.3. Construction of Protein–Protein Interaction (PPI) Network and Molecular Modules

The PPI network of coexpressed DEGs was constructed in the STRING database (https://string-db.org/), and the default settings recommended by the database were used; furthermore, the meaning of network edges was set as “confidence,” and, finally, the display was to intended to hide disconnected nodes in the network [24]. We downloaded the PPI–data and reconstructed gene module network, containing functional gene modules and their interactions using the MCODE plug-in Cytoscape software (version 3.7.1, https://cytoscape.org/). In MCODE, filters were based on the default parameters “Degree Cutoff = 2,” “Node Score Cutoff = 0.2,” “K − Core = 2,” and “Max.Depth = 100” [25]. Genes contained in the most important molecular modules were identified as key genes.

### 2.4. Gene Ontology (GO) and Kyoto Encyclopedia of Genes and Genomes (KEGG) Pathway Analyses

While the GO annotation (biological process (BP), cellular component (CC), and molecular function (MF)) are independent of each other, in essence, there remains significant crosstalk, which, together with KEGG pathway enrichment analysis, is also used to evaluate the biological effects of genes [26]. In this study, we performed GO and KEGG pathway analyses for key genes using the clusterProfiler package [27]. And the results were visualized by bar plot. Adjusted *P* < 0.05 was the selecting criterion.

### 2.5. Relationships between the Key Genes' Expression and Clinicopathologic Characteristics of HCC

Using the Oncomine database, we selected three publicly available data sets to for our validation effort: Chiang Liver [28], Jia Liver [29], and Wurmbach Liver [30]. With Chiang Liver, we evaluated the expression levels of key genes in TP53 status. With Jia Liver, we explored the gene expression level in different stage besides tumor size of HCC. In Wurmbach Liver, we observed the gene expression level in varying states of liver disease, tumor grade, tumor size, and vascular invasion. Statistical analysis was performed to determine differences between groups, and the results were illustrated using box and scatter plots prepared in *R* software.

### 2.6. Prognosis Analysis

Survival analysis was conducted using the GEPIA (http://gepia.cancer-pku.cn/index.html) [31] and the Kaplan–Meier plotter (http://kmplot.com/analysis/) [32] databases so as to assess the impact of key genes on the survival time (OS and disease-free survival (DFS) were assessed in GEPIA, and recurrence-free survival (RFS) and progress-free survival (PFS) were evaluated by the Kaplan–Meier plotter database). The results were visualized by survival curve and forest plot (https://cran.r-project.org/web/packages/forestplot), respectively. Meanwhile, the hazard ratio (HR), 95% confidence interval (95% CI), and log-rank *P* value were calculated and described.

### 2.7. Analyses of Key Gene—Tumor Microenvironment (TME) Interactions

As reported, hypoxia, genetic instability, and immune evasion become key features of the liver microenvironment [33]. Hence, we evaluated not only the correlations of key genes with infiltrated cells in the TME of HCC but also association with hypoxic signatures. Based on the TIMER database [34, 35], we first used the xCell method to investigate the impact of immune cells (B cells, T cells, neutrophils, dendritic cells, hematopoietic stem cells (HSCs), and natural killer cells) and stromal cells (endothelial cells (ECs)) in the TME on the OS of HCC patients and selected the tumor immune dysfunction and exclusion (TIDE) method to evaluate the impact of M2-type tumor-associated macrophages (M2-TAM), myeloid-derived suppressor cells (MDSCs), and cancer-associated fibroblasts (CAFs) on the survival of HCC patients. We then obtained the cell types correlated with poor prognosis in HCC patient, which we called prognosis-related cells. Second, we investigated the correlations between key genes and prognosis-related cells. Third, from the GEPIA and TIMER databases, we detected correlations between the key genes and biomarkers of prognosis-related cells reported by previous research (CD11B, CD33 for MDSCs, CD34, CD117 for HSCs, CD31, CD105 for ECs) [36–38] and hypoxic microenvironment-forming factors such as hypoxia-inducible factor 1*α* (HIF1A) and VEGFA [39]. The results were visualized using the corrplot *R* package.

### 2.8. Statistical Analysis

R 3.6.2 and GraphPad Prism were used for statistical analysis. The D'Agostino-Pearson normality test was used to describe the distribution of the gene expression. The *F*-test was used to evaluate the homogeneity of variance. Student's *t*-test, one-way ANOVA, and the Mann–Whitney-Wilcoxon test were used to determine the statistical significance between groups according to data distribution and numbers of compared groups. Kaplan–Meier analysis and the log-rank test were applied to determine the survival curves. Correlations between key genes, infiltrating cell types, and gene markers were established by Spearman's correlation, and correlation strength was classified according to the absolute value of the partial correlation coefficient as follows: 0.00-0.19 “a negligible correlation”; 0.20-0.39 “a weak correlation”; 0.40-0.59 “a moderate correlation”; 0.60-0.79 “a strong correlation”; and 0.80-1.0 “a very strong correlation” [40, 41]. The results were considered to have statistical significance when *P* < 0.05. Survival curves were obtained from the GEPIA and the Kaplan–Meier plotter databases and displayed with HR and *P* values from the log-rank test.

## 3. Results

### 3.1. Microarray Data

**Figure 1:**
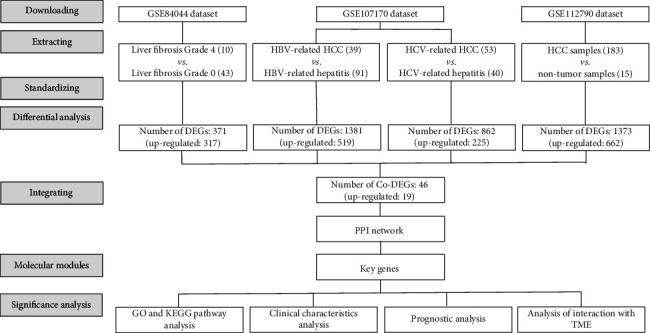
The data processing workflow. HBV: hepatitis B virus; HCV: hepatitis C virus; HCC: hepatocarcinoma; DEGs: differentially expressed genes; PPI: protein-protein interaction; GO: gene oncology; KEGG: Kyoto Encyclopedia of Genes and Genomes; TME: tumor microenvironment.

From the GSE84044 data set, we extracted gene expression data of patients with liver fibrosis of grade 0 (no fibrosis, 43 samples) and grade 4 (early cirrhosis, 10 samples). Furthermore, from the GSE107170 data set, we selected data from tumor (HBV- or HCV-related HCC) and nontumor samples (HBV- or HCV-related hepatitis), and from the GSE112790 data set, we used the whole data. Finally, four sets of data were obtained, and the details of data preparation and processing are given in Figure 1. All candidate data sets were normalized, and the results are shown in Figure S1.

### 3.2. Identification of DEGs

**Figure 2:**
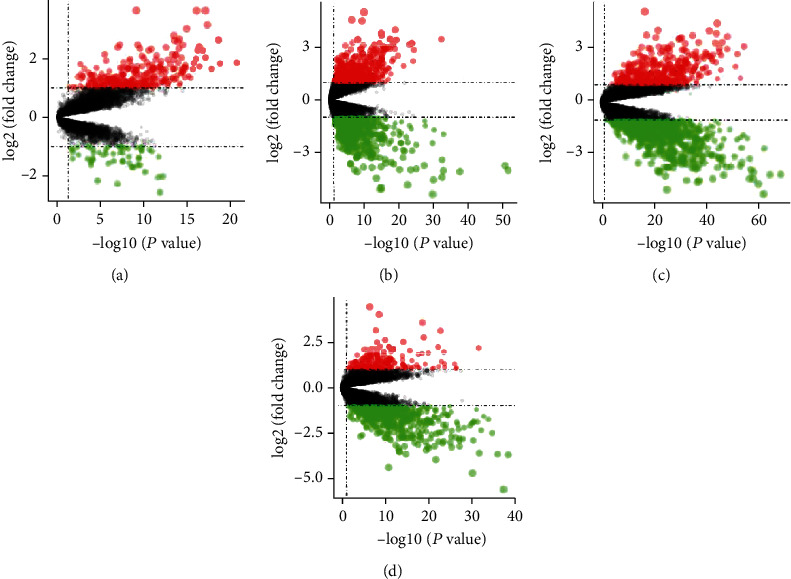
Differential analyses of the gene expression profile data. (a)–(d) Volcano plot for differential expression genes in GSE84044 data set (a), GSE112790 data set (b), HBV data from GSE107170 data set (c), and HCV data from GSE107170 data set (d). Red point represents a gene with log fold change > 1 and *P* < 0.05, green point represents a gene with log fold change < −1 and *P* < 0.05, and black point represents a gene with −1 < log fold change < 1 and *P* > 0.05. Dashed lines in *y* axis were for position of log fold change = 1 and -1, and dashed line in *x* axis is for position of *P* = 0.05.

**Figure 3:**
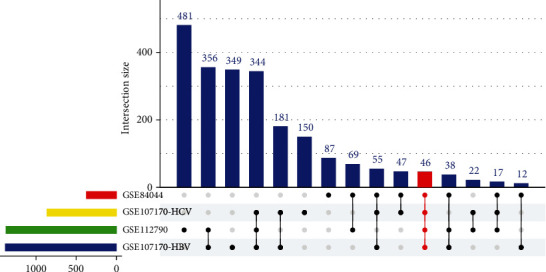
The number of coexpressed DEGs in the candidate datasets. HBV: hepatitis B virus; HCV: hepatitis C virus.

The DEGs from four sets of data are shown in Figures 2(a)–2(d). Forty-six coexpressed DEGs were obtained by integrating bioinformatic analysis (Figure 3), covering 19 upregulated expression genes and 26 downregulated expression genes. The details of the coexpressed DEGs are provided in supplementary Table S1.

### 3.3. PPI Network and Molecular Modules

**Figure 4:**
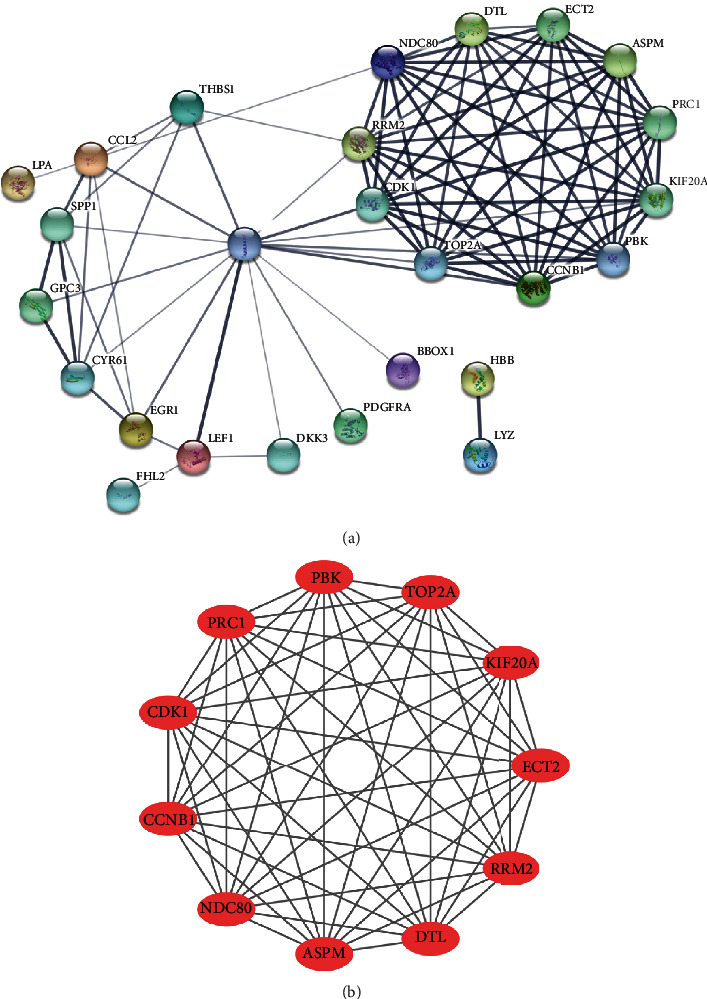
Protein-protein interaction (PPI) network for the coexpressed differential expression genes (DEGs) from the STRING database and the most important molecular module constructed by Cytoscape software. (a) PPI network and lines represent interactions, and the degree of thickness represents strength of evidences. (b) the most important molecular module and lines represent interactions, and the red represents upregulated gene.

The PPI network for coexpressed DEGs was built through the STRING database including 26 nodes and 89 edges, and the result are shown in Figure 4(a). Two molecular modules were identified using MCODE, the most important of which contained 11 key genes (RRM2, NDC80, ECT2, CCNB1, ASPM, CDK1, PRC1, KIF20A, DTL, TOP2A, and PBK), as visualized in Figure 4(b), which also revealed that this molecular module plays an important role in the process of hepatocarcinogenesis.

### 3.4. Functional Enrichment and KEGG Pathway Analyses for Key Genes

**Figure 5:**
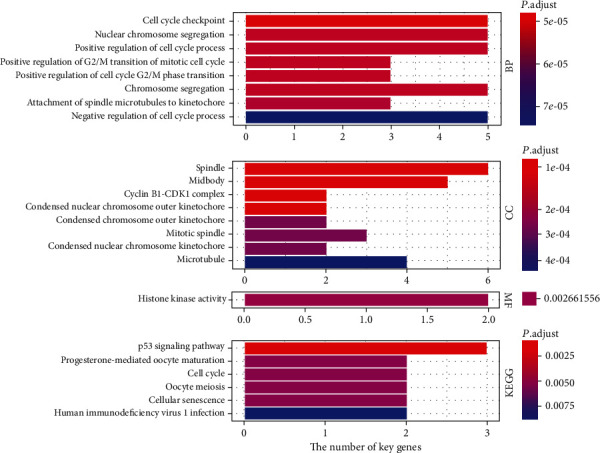
Bar plot for enriched biological process (BP), cellular component (CC), molecular function (MF), and KEGG pathways for coexpressed key gene.

The results of GO function and KEGG pathway analyses for 11 key genes were shown in Figure 5. It was evidence that these genes were mainly involved in the BPs of the cell cycle such as cell cycle checkpoint, chromosome segregation, histone phosphorylation, and the CCs of the cyclin-dependent protein kinase holoenzyme complex and molecular functions of cyclin-dependent protein kinase activity. Beyond that, KEGG pathway enrichment was mainly focused on p53 and the cell cycle signaling pathway. These results suggested that the key genes largely belong to cell cycle-related genes, which also highlighted signaling of the cell cycle that contributes to the tumor growth of HCC, which may be a potential therapeutic target for HCC patients.

### 3.5. Relationships between the Expression of Key Genes and Clinicopathologic Characteristics of HCC

**Figure 6:**
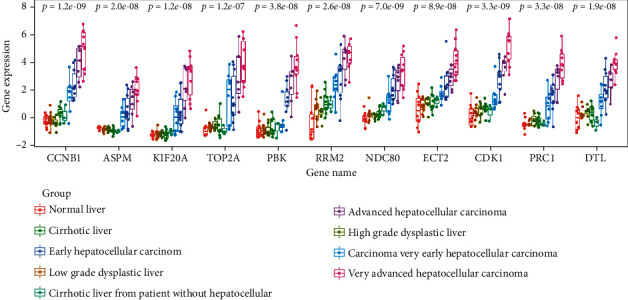
Different expression levels of key genes in various liver diseases based on Wurmbach Liver data set from the Oncomine database.

**Figure 7:**
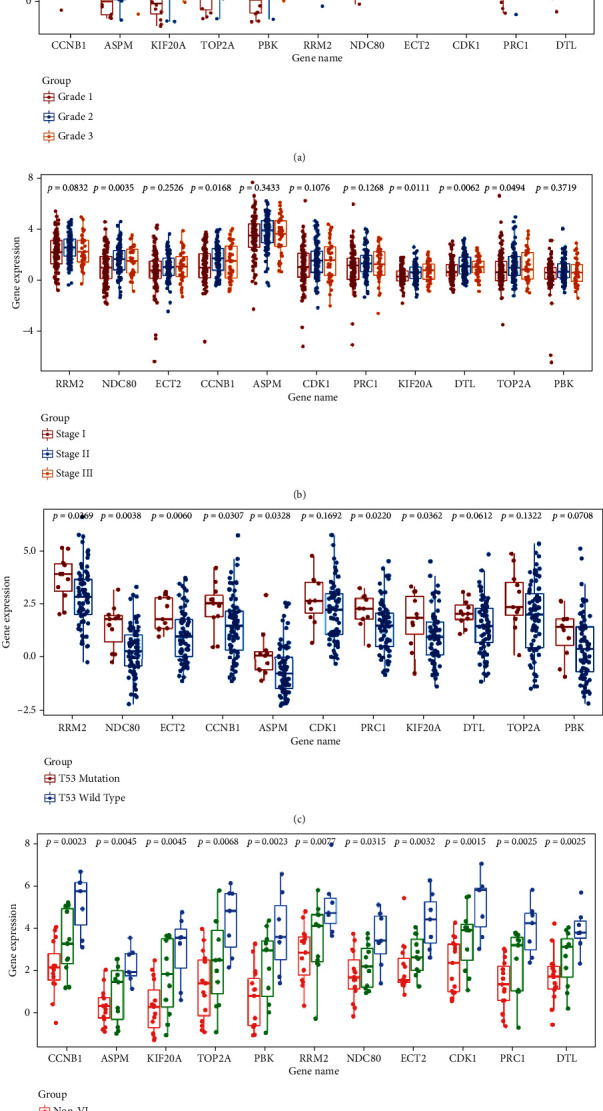
Different expression levels of key genes between various clinical characteristics of hepatocellular carcinoma (HCC) patients. (a) Key genes' expression in grades 1-3 of patients with HCC in Wurmbach Liver data set. (b) Key genes' expression in stages I-III of patients with HCC in Jia Liver data set. (c) Key genes' expression in the different status of TP53 mutation of patients with HCC in Chiang Liver data set. (d) Key genes' expression in the different statuses of vascular invasion of patients with HCC in Wurmbach Liver data set. TP53: tumor protein p53; VI: vascular invasion.

For three data sets (Wurmbach Liver, Chiang Liver, and Jia Liver) from the Oncomine database, the expression levels of 11 key genes in the different states of liver diseases showed obvious differences in Wurmbach Liver data set (*P* < 0.05, Figure 6). And the results indicated that all key genes had a low expression level in nontumor liver tissues but a high level in HCC tissues, which suggested that these key genes play a vital role in hepatocarcinogenesis. Besides, in this data set, we also observed a low expression level of the key genes in grade 1 but a high level in grades 2 and 3 and significant differences among the three groups (*P* < 0.05, Figure 7(a)). For tumor stage, significant differences among stage 1, stage 2, and stage 3 were shown in NDC80, CCNB1, KIF20A, DTL, and TOP2A of the key genes from Jia Liver data set (*P* < 0.05, Figure 7(b)). In addition, significant differences (*P* < 0.05, Figure 7(c)) between the TP53 mutation and wild type were also observed in the partial key genes except CDK1, DTL, TOP2A, and PBK in Chiang Liver data set. Interestingly, in HCC samples with vascular invasion, especially macroscopic ones, we found a high expression level of key genes, and there was significant difference (*P* < 0.05, Figure 7(d)) compared to samples without vascular invasion according to Wurmbach Liver data set. However, the differences among tumor size (diameter ≥ 3 cm vs. diameter < 3 cm; diameter ≥ 5 cm vs. diameter < 5 cm) did not reach statistical significance (Figure S2). These results further enriched the evidence that the key genes are involved in the initiation and progression of HCC.

### 3.6. Prognosis Analysis

**Figure 8:**
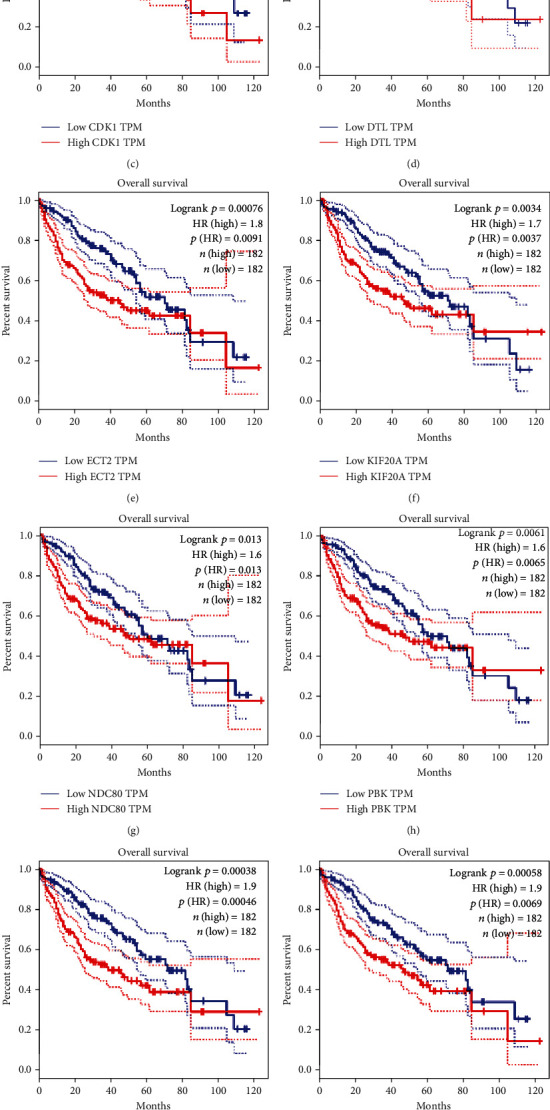
Overall survival (OS) curves (a)–(k) comparing the high and low expression of key genes in patients with hepatocellular carcinoma (HCC) from the GEPIA database. HR: hazard ratio.

**Figure 9:**
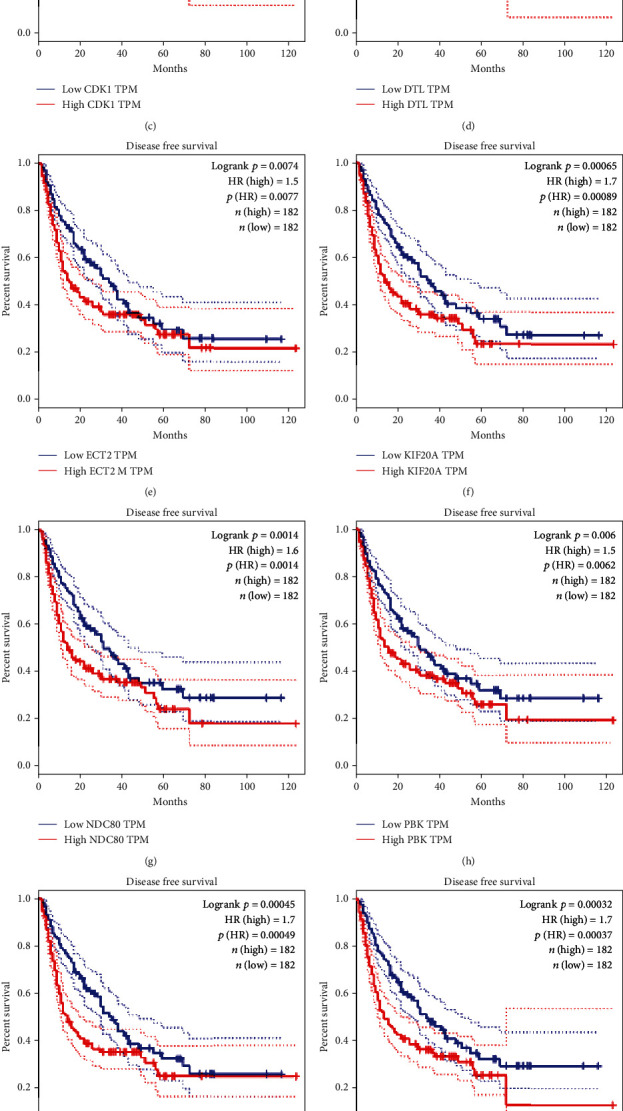
Disease-free survival (DFS) curves (a)–(k) comparing the high and low expression of key genes in patients with hepatocellular carcinoma (HCC) from the GEPIA database. HR: hazard ratio.

**Figure 10:**
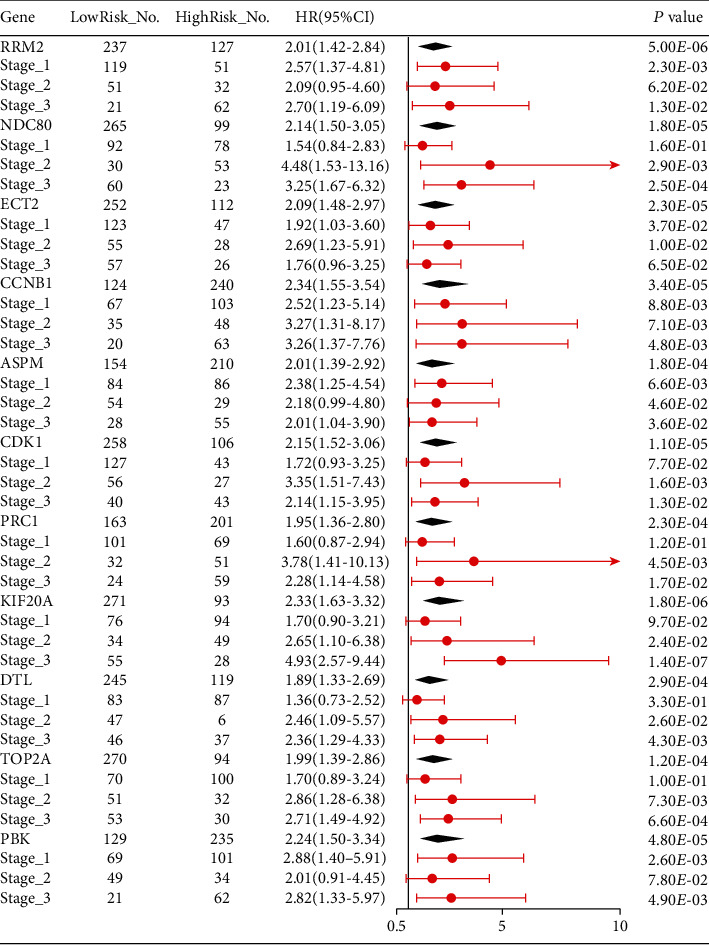
Forest plot for overall survival (OS) comparing the high and low expression of key genes in various stages based on the Kaplan Meier-Plotter database. NO.: the number of patients with gastric cancer; 95% CI: 95% confidence interval; HR: hazard ratio.

**Figure 11:**
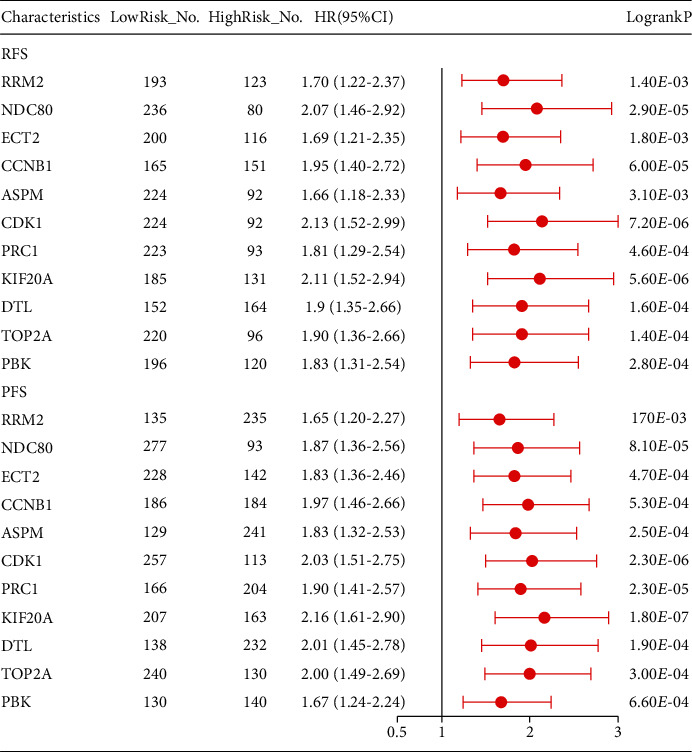
Forest plot for recurrence-free survival (RFS) and progress-free survival (PFS) comparing the high and low expression of key genes in hepatocarcinoma based on the Kaplan Meier-Plotter database. NO.: the number of patients with gastric cancer; 95% CI: 95% confidence interval; HR: hazard ratio.

From the GEPIA database, it turned out that the high level of 11 key gene expressions was both associated with poor OS (Figure 8) and DFS (Figure 9) of HCC patients, which was similar to the results from the Kaplan–Meier plotter database (Figure 10). Furthermore, we also found that the high expression of NDC80, CCNB1, CDK1, PRC1, KIF20A, DTL, and TOP2A was significantly associated with worse OS in advanced T-stage (T2–3) patients (Figure 10). The outcomes of RFS and PFS also showed that the high expression of these key genes had a significant association with poor prognosis in the cohort of HCC patients, as shown in Figure 11. Collectively, these data showed that the high key gene expression promotes HCC progression and indicates poor prognosis.

### 3.7. Key Gene—TME Interactions

**Figure 12:**
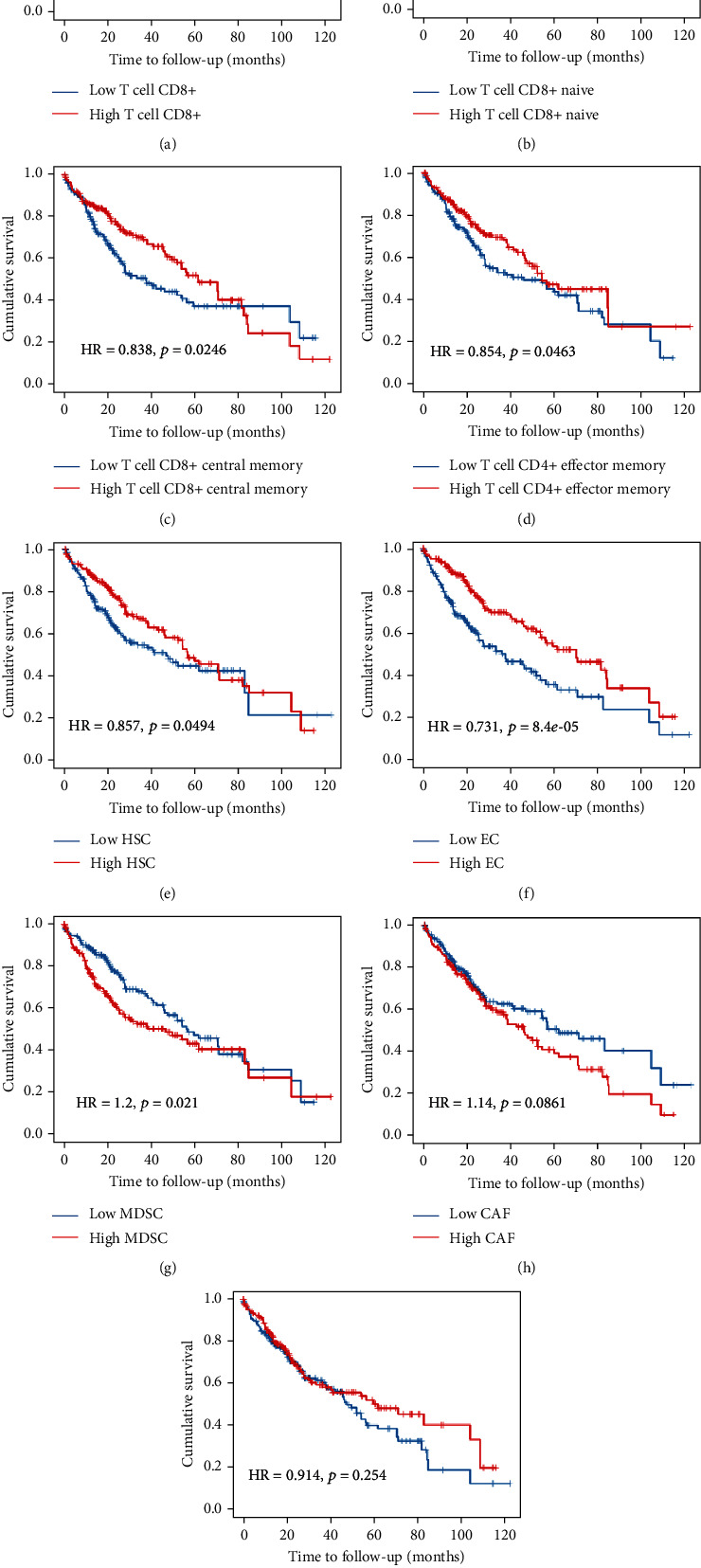
Kaplan-Meier survival curves comparing various immune cells in the tumor microenvironment (TME) of hepatocarcinoma based on the TIMER database. HR: hazard ratio.

**Figure 13:**
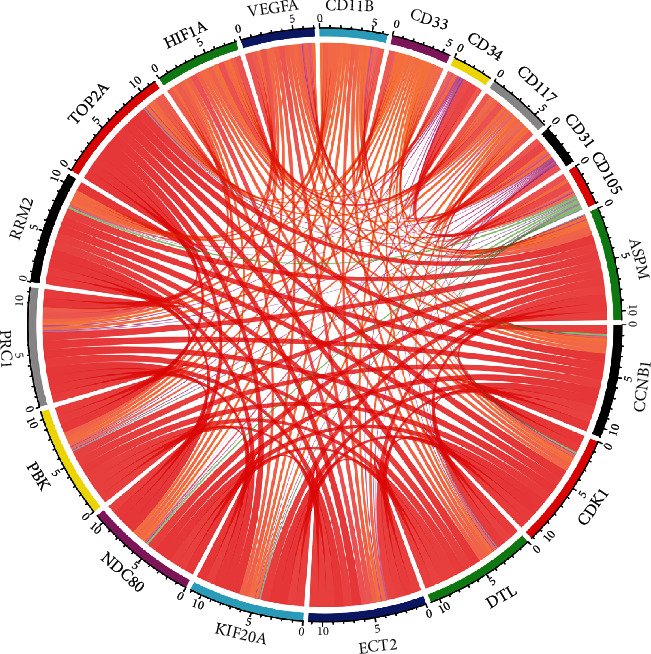
The correlations between key genes and biomarkers of immune cells and hypoxic signature (CD11B and CD33 for MDSCs, CD34 and CD117 for HSCs, CD31 and CD105 for ECs, and HIF1A and VEGFA for hypoxia). Dark red line represents the strength of correlation greater than 0.6, light red line represents the strength of correlation between 0.3 and 0.6, purple line represents the strength of correlation less than 0.3, and green line represents the strength of correlation between -0.3 and 0.

From the TIMER database, we analyzed the immune cell types in the TME of HCC and found that the infiltrated levels of the T cell family of T cell CD8+, CD8+ naive, CD8+ central memory, and CD4+ effector memory were associated with good prognosis of HCC. In addition, the infiltrated levels of HSC and EC were found to significantly predict a better prognosis of HCC patients. However, using the TIDE method, we found that the high level of MDSC infiltration indicated a worse prognosis in patients with HCC, and the details are shown in Figure 12. Correlations with prognosis-related infiltrated cells showed that there was a positive association of key genes with MDSCs but negative regulation with HSCs and ECs. However, no significant correlations were evident for the T cell family (Table 1). Similar results were also seen for the correlation analysis between key genes and biomarkers of MDSCs, ECs, HSCs, and hypoxia in the TIMER database (Figure 13) and the GEPIA database (Table 2).

## 4. Discussion

HCC is a highly complex heterogeneous tumor [42], which gradually occurs on the basis of chronic liver disease through the gathering of different genomic alterations, and its prognosis is also closely related to the multistep process of underlying liver disease [43]. From a clinical perspective, detection, characterization, and identification of appropriate treatment strategies and improvement of HCC prognosis have always been the major concerns clinically [44]. Thus, to improve the early diagnostic rate, the identification of sensitive and specific prognostic biomarkers is of great importance.

In our study, with the differential analysis of microarray data from the GEO database, 11 cell cycle-related key genes (RRM2, NDC80, ECT2, CCNB1, ASPM, CDK1, PRC1, KIF20A, DTL, TOP2A, and PBK) involved in the process of the transition from liver cirrhosis to carcinoma were identified. With the Oncomine database, we also found that the key genes were presented at high levels in advanced HCC samples and HCC with vascular invasion. Meanwhile, prognostic analysis showed that the key genes were significantly correlated with poor prognosis of HCC patients using the Kaplan–Meier plotter and GEPIA databases. Subsequently, we evaluated the effect of key genes on the TME of HCC. The results demonstrated that the key genes were positively correlated with MDSCs that infiltrated in the TME of HCC and led to poor prognosis, but negatively correlated with ECs and HSCs that were associated with good prognosis. Molecular oxygen plays a unique role in the cell cycle, cell growth, and cell energy metabolism [45]. Hence, we further evaluated the influence of key genes on the hypoxic signatures (HIF1A and VEGFA) of HCC tissues, and the results showed that there was a positive correlation between them. In all, our findings demonstrated that the key genes might promote liver cirrhosis and HCC progression and tended to indicate a poor prognosis, which might be largely due to the consequence of the key genes driving the communications between tumor cells and the TME and accelerating the formation of a hypoxic and immunosuppressive microenvironment.

Gene modules enable better understanding of molecular mechanisms of disease progression, as a module is usually defined as a group of coexpressed genes or genes with a joint role [46]. In our study, we analyzed the differential gene expression patterns in liver cirrhosis and HCC caused by varying liver disease and found that the most important molecular module consisted of 11 key genes, which provided us with a new understanding of genes that have a role both in the development of liver cirrhosis and HCC. For gene functions, enrichment analysis showed that the key genes are a highly cell cycle-related gene set, as the GO annotation was enriched in cell cycle checkpoint, chromosome segregation, positive regulation of cell cycle process, and histone phosphorylation, and the KEGG pathway was enriched in TP53 and cellular senescence signaling. Moreover, we also identified that there is a high level of expression of key genes in the HCC sample with a TP53 mutation. As reported, TP53-regulated genes are involved in diverse biological pathways including the cell cycle, DNA damage response, apoptosis, and glucose metabolism [47]. The cell cycle is mainly regulated by a series of cyclins, cyclin-dependent kinases (CDKs), and cyclin-dependent kinase inhibitors (CDKIs) [48]. Deregulated cell cycle progression is a hallmark of human cancer, and targeting CDKs to block cell proliferation has been validated as an effective anticancer therapy [49]. Based on this, our results presented potential targets for the treatment of HCC patients. From a clinical perspective, vascular invasion and metastasis are major challenge for current HCC treatment, and macrovascular and microvascular invasion are also indicators for poor prognosis in HCC patients [50]. In our analysis, the key genes were found to be overexpressed in advanced HCC patients and in those with a higher grade status of HCC, which is particularly more apparent in the tissues of HCC with vascular invasion. Furthermore, these observations provided valuable insights into the identification of potential prognostic biomarkers for HCC patients.

Most of 11 key genes have been reported to be prognostic biomarkers of HCC by previous research [4, 51–56]; however, there has been little discussion on the relationship with the TME of HCC. In addition, through cell cycle signaling, it is reasonable to explain how HCC occurs, but explanations as to why key genes caused poor prognosis in HCC patients have not been convincing. Thus, we next turned our attention to the TME of HCC. During cancer development, the TME, with infiltrating immune and nonimmune cells, and the extracellular matrix undergo substantial changes that can influence tumor progression [57, 58]. Furthermore, hypoxia is also a hallmark of the TME [59], and it has been reported to play an important role in the development of liver diseases [60, 61]. Hence, to further survey the influence of the key genes on the prognosis of HCC, we investigated their interactions with tumor-infiltrating cells and hypoxic signatures. For analysis methods, xCell, a compendium of newly generated gene signatures for 64 cell types, is frequently used to evaluate the infiltrated levels of immune and stromal cells [62]. TIDE, an accurate gene signature to model tumor immune escape, is generally used to identify the gene expression signature of T cell exclusion [63]. In our study, we examined the infiltration of three types immune cells (CAFs, MDSCs, and M2-TAMs) using the TIDE method, as it has been reported that these three types of cells restrict cytotoxic T cell infiltration in tumors [64]; we used the xCell method to evaluate other infiltrating cells. From the results, although we found that the T cell family is associated with good prognosis of HCC, there was no direct evidence confirming that the key genes had significant correlation with them. Interestingly, we found that ECs belonging to the stromal cell type infiltrated in the TME were also associated with better prognosis of HCC, and the 11 key genes had significantly negative correlations with it. For HCC with specific tissue characteristics and a special blood supply, high-infiltrating ECs led to patients obtaining a better prognosis, which might be theoretically explained by the presence of an endothelial barrier. Given that Strilic et al. reported that tumor cell-induced EC necroptosis promotes metastasis [65], the endothelial barrier formed by EC infiltration into the TME may be critical for maintaining the stabilization of the microenvironment. Abnormal expression of key genes by tumor cells might lead to the destruction of the endothelial barrier and attenuate the blocking effect of ECs on tumor cells, which allows tumor cells to more easily invade adjacent blood vessels. This might be a potential route of HCC-developed intrahepatic metastasis. However, more evidence is needed to confirm this hypothesis.

For another, HSCs are involved in the proliferation and repair of hepatocytes [66, 67], which is essential to maintain the normal physiological characteristics of liver cells. In our analysis, we found that the key genes had a negative correlation with the infiltrated level of HSCs in the HCC tissues, which indicated that when liver cancer occurs, the abnormal expression of key genes significantly reduces the number of HSCs in the microenvironment, which weakens the ability of HSCs to repair damaged liver cells, and, to some extent, turns the microenvironment into a cancer-promoting state.

MDSCs rapidly expand during inflammation, infection, and cancer [68]; however, previous research described that the increase of MDSCs was not correlated with hepatic fibrosis or the disease activity of chronic liver disease [69]. For HCC, not only did we find that the infiltrated level of MDSCs correlated with poor prognosis of HCC, which is in line with data reported in the literature [70–72], but also that there was a significantly positive correlation with key genes. As mentioned before, MDSCs are unable to stimulate an allogeneic T cell response and suppress T cell proliferation [3]. Therefore, although we found that the T cell family had a good prognosis for patients with HCC, this small advantage would be negated by the MDSCs because tumor cells with high expression of key genes could recruit more MDSCs into the microenvironment and promote the formation of a tumor immunosuppressive environment. Furthermore, the role of hypoxia in the progression of liver disease and even HCC has been confirmed [73–75]. Recent studies show that hypoxia-induced genes play an important role in the diagnosis and treatment of liver cancer [76–78] and are a factor that seriously influences the efficacy of sorafenib [79–81]. In our study, we explored the relationship between the key genes and the HIF-1*α* (HIF1A) factor and its targeted gene (VEGFA), and the results indicated that there was a positive correlation between them. These pieces of evidence suggested that the aberrant expression of key genes is largely dependent on the hypoxia status of tumor cells, and that the key genes may be hypoxia-inducible genes.

Based on the available evidence, we can attempt to explain the underlying molecular mechanisms of key genes involved in tumorigenesis and poor prognosis of HCC. Normal liver cells were repeatedly stimulated by undesirable interfering factors (such as hepatitis virus, alcohol, etc.), they interacted with hypoxia and other factors in the cell microenvironment, and they gradually induced abnormal expression of key genes, which led to the cell cycle dysregulation that is essential for cellular transformation [82]. Disorders of the cell cycle allow liver cells to acquire the ability to become cancerous. When liver cells become cancer cells, the abnormal expression of the key genes further intensifies, and moreover, induces the aggregation of MDSCs into the TME. The infiltration of MDSCs prevents cytotoxic T cells from entering tumor tissue and allows tumor cells to escape the immune response. At the same time, the aberrant expression of the key genes by tumor cells negatively regulated the ECs infiltrating in the TME, causing damage of ECs function, destruction of the endothelial barrier, and homeostasis imbalance of the microenvironment. Furthermore, one additional point merits further concern, which is that the abnormal expression of key genes impairs the ability of HSCs to repair damaged liver cells, and, to a certain extent, also promotes the cancerization of liver cells. These biological effects connect with each other and are influenced by each other, and they provide a suitable growth environment for cancer cell. However, they eventually allow liver cancer cells to make an immune escape and gain the ability of sustained progression.

However, many questions remain. Does the EC barrier really exist in the TME of HCC? How the relationship between the EC barrier and VEGFA be verified? Based on our study, key genes are negatively correlated with ECs but positive correlation with VEGFA. Thus, for HCC, is there a special relationship between VEGFA and ECs? Are the abnormal expressions of key genes really the outcomes of hypoxia induction? How do the key genes recruit MDSCs to enter the TME and promote the formation of the immunosuppressive microenvironment? Our findings provided novel clues that now require indepth analysis of these problems by means of further experiments.

## 5. Conclusions

In conclusion, we identified 11 key genes (RRM2, NDC80, ECT2, CCNB1, ASPM, CDK1, PRC1, KIF20A, DTL, TOP2A, and PBK) that may play a vital role in the pathogenesis of HCC and drive the communication between tumor cells and the TME and act as a promising diagnostic, therapeutic, and prognostic biomarker in HCC patients.

## Figures and Tables

**Table 1:** The correlations of key genes with the T cell family, hematopoietic stem cell (HSCs), endothelial cells (ECs), and myeloid-derived suppressor cells (MDSCs). Cor: coefficient of partial correlation from the TIMER database.

Gene	T cell CD8+		T cell CD8+ naive		T cell CD8+ central memory		T cell CD4+ effector memory		HSCs		ECs		MDSCs	
	Cor	*P* value	Cor	*P* value	Cor	*P* value	Cor	*P* value	Cor	*P* value	Cor	*P* value	Cor	*P* value
*RRM2*	0.108	4.47*E*-02	-0.079	1.44*E*-01	0.08	1.40*E*-01	-0.127	1.87*E*-02	-0.505	1.01*E*-23	-0.513	1.65*E*-24	0.638	8.42*E*-41
*NDC80*	0.066	2.21*E*-01	-0.072	1.83*E*-01	0.038	4.82*E*-01	-0.141	8.87*E*-03	-0.474	9.03*E*-21	-0.511	2.44*E*-24	0.692	1.85*E*-50
*ECT2*	0.001	9.84*E*-01	-0.206	1.15*E*-04	-0.017	7.57*E*-01	-0.098	6.82*E*-02	-0.423	1.93*E*-16	-0.496	7.70*E*-23	0.673	7.47*E*-47
*CCNB1*	0.041	4.52*E*-01	-0.124	2.09*E*-02	-0.011	8.43*E*-01	-0.148	6.03*E*-03	-0.515	8.65*E*-25	-0.527	5.16*E*-26	0.735	8.23*E*-60
*ASPM*	0.052	3.37*E*-01	-0.079	1.42*E*-01	0.012	8.26*E*-01	-0.149	5.67*E*-03	-0.393	3.28*E*-14	-0.433	3.12*E*-17	0.598	7.40*E*-35
*CDK1*	0.066	2.23*E*-01	-0.096	7.56*E*-02	0.014	7.93*E*-01	-0.153	4.50*E*-03	-0.463	9.37*E*-20	-0.508	4.68*E*-24	0.718	7.81*E*-56
*PRC1*	-0.01	8.56*E*-01	-0.155	3.99*E*-03	-0.029	5.95*E*-01	-0.159	3.01*E*-03	-0.420	3.70*E*-16	-0.483	1.44*E*-21	0.683	1.22*E*-48
*KIF20A*	0.045	4.01*E*-01	-0.106	4.91*E*-02	-0.024	6.62*E*-01	-0.186	5.22*E*-04	-0.477	5.72*E*-21	-0.489	3.56*E*-22	0.712	1.46*E*-54
*DTL*	0.026	6.25*E*-01	-0.104	5.27*E*-02	0.001	9.83*E*-01	-0.18	7.98*E*-04	-0.403	6.21*E*-15	-0.478	4.65*E*-21	0.65	1.01*E*-42
*TOP2A*	0.043	4.21*E*-01	-0.104	5.27*E*-02	0.006	9.06*E*-01	-0.157	3.43*E*-03	-0.434	2.82*E*-17	-0.486	7.36*E*-22	0.669	3.56*E*-46
*PBK*	0.045	4.06*E*-01	-0.094	8.23*E*-02	0.014	7.93*E*-01	-0.155	4.01*E*-03	-0.393	3.29*E*-14	-0.433	3.66*E*-17	0.625	9.40*E*-39

**Table 2:** The correlations of key genes with biomarkers of immune cells and hypoxic signature (CD11B and CD33 for MDSCs, CD34 and CD117 for HSCs, CD31 and CD105 for ECs, and HIF1A and VEGFA for hypoxia). R: Spearman correlation coefficient from the GEPIA database.

Gene	*HIF1A*		*VEGFA*		*CD11B*		*CD33*		*CD34*		*CD117*		*CD31*		*CD105*	
	R	*P* value	R	*P* value	R	*P* value	R	*P* value	R	*P* value	R	*P* value	R	*P* value	R	*P* value
*ASPM*	0.36	4.60*E*-13	0.43	2.00*E*-18	0.22	1.40*E*-05	0.16	2.10*E*-03	0.033	5.20*E*-01	0.2	7.80*E*-05	0.08	1.20*E*-01	-0.20	1.30*E*-04
*CCNB1*	0.30	3.70*E*-09	0.33	5.60*E*-11	0.27	8.70*E*-08	0.23	1.10*E*-05	-0.07	1.80*E*-01	0.12	2.40*E*-02	-0.02	6.90*E*-01	-0.28	4.80*E*-08
*CDK1*	0.37	1.40*E*-13	0.44	1.10*E*-18	0.26	2.40*E*-07	0.18	6.60*E*-04	-0.01	8.80*E*-01	0.21	5.40*E*-05	0.05	3.30*E*-01	-0.23	5.60*E*-06
*DTL*	0.45	9.00*E*-20	0.50	2.50*E*-24	0.35	2.10*E*-12	0.15	4.40*E*-03	0.05	3.10*E*-01	0.22	1.70*E*-05	0.11	3.20*E*-02	-0.15	1.10*E*-03
*ECT2*	0.58	2.90*E*-34	0.57	3.40*E*-33	0.40	8.20*E*-16	0.22	1.60*E*-05	0.09	7.40*E*-02	0.34	2.20*E*-11	0.19	3.00*E*-04	-0.08	1.10*E*-01
*KIF20A*	0.40	9.00*E*-16	0.46	6.50*E*-21	0.31	2.00*E*-09	0.17	1.20*E*-03	0.01	9.10*E*-01	0.24	2.30*E*-06	0.06	2.10*E*-01	-0.21	5.40*E*-05
*NDC80*	0.33	4.20*E*-11	0.40	5.60*E*-16	0.30	2.40*E*-09	0.22	1.70*E*-05	-0.03	5.60*E*-01	0.14	6.10*E*-03	0.02	7.10*E*-01	-0.23	8.00*E*-06
*PBK*	0.42	2.60*E*-17	0.42	2.80*E*-17	0.30	6.80*E*-09	0.16	1.80*E*-03	0.06	2.50*E*-01	0.23	1.10*E*-05	0.12	1.90*E*-02	-0.17	1.40*E*-03
*PRC1*	0.34	2.10*E*-11	0.46	4.60*E*-21	0.28	3.50*E*-08	0.16	1.60*E*-03	-0.03	5.70*E*-01	0.17	1.00*E*-03	0.017	7.50*E*-01	-0.22	1.80*E*-05
*RRM2*	0.43	1.20*E*-17	0.45	1.40*E*-19	0.37	1.10*E*-13	0.24	4.50*E*-06	0.04	4.90*E*-01	0.22	2.90*E*-05	0.14	7.10*E*-03	-0.19	3.10*E*-04
*TOP2A*	0.17	9.40*E*-04	0.29	1.50*E*-08	0.21	5.80*E*-05	0.19	3.50*E*-04	-0.15	3.30*E*-03	0.03	5.70*E*-01	-0.13	1.10*E*-02	-0.34	2.20*E*-11

## Data Availability

The datasets analyzed in this study are available in the Oncomine (https://www.oncomine.org), GEO (https://www.ncbi.nlm.nih.gov/geo/), TIMER (https://cistrome.shinyapps.io/timer/), and GEPIA (http://gepia.cancer-pku.cn/index.html) databases.

## Additional Points

*Standard Biosecurity and Institutional Safety Procedures*. We adhered to standard biosecurity and institutional safety procedures of Lanzhou University Second Hospital, Gansu Provincial Hospital, and Southern University of Science and Technology Hospital.

## Supplementary Materials

Supplementary Materials Supplementary 1. Figure S1: normalization of the gene expression. Supplementary2. Figure S2: different expression levels of key genes between various tumor sizes of hepatocellular carcinoma (HCC) patients. Supplementary 3. Table S1: the coexpressed differentially expressed genes from candidate datasets.
