# Supplementary material for: Bioinformatic Evidence Reveals that Cell Cycle Correlated Genes Drive the Communication between Tumor Cells and the Tumor Microenvironment and Impact the Outcomes of Hepatocellular Carcinoma
Source: Biomed Res Int. 2021 Oct 26;2021:4092635. doi: 10.1155/2021/4092635 (PMC8564189; doi:10.1155/2021/4092635)
Supplement: Supplementary Materials — Supplementary 1. Figure S1: normalization of the gene expression. Supplementary2. Figure S2: different expression levels of key genes between various tumor sizes of hepatocellular carcinoma (HCC) patients. Supplementary 3. Table S1: the coexpressed differentially expressed genes from candidate datasets. [file 4092635.f1.pdf]

Supplementary Material

1 Supplementary Figures and Tables

1.1 Supplementary Figures

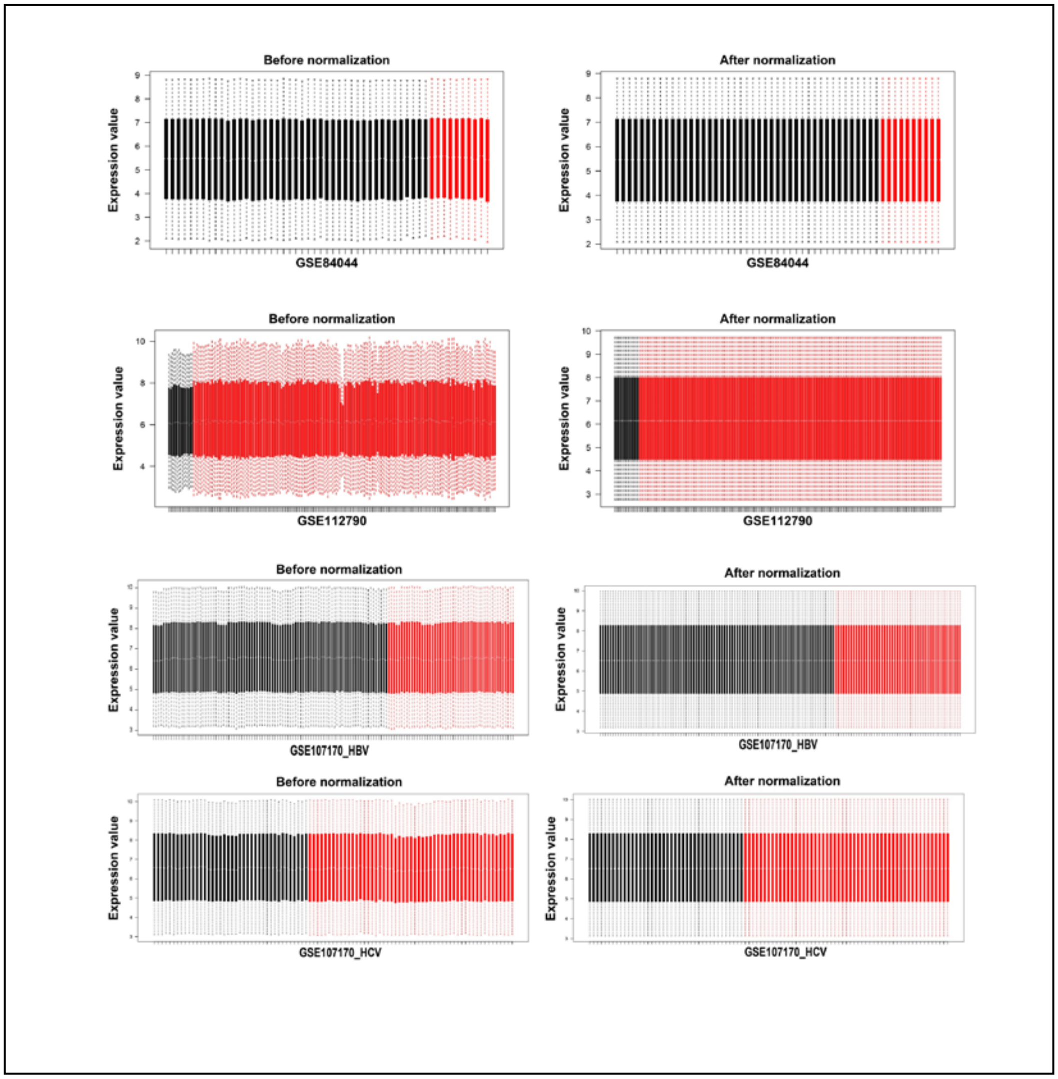

Supplementary Figure S1. Normalization of gene expression. Red represents hepatocellular carcinoma tissues and black represents nontumor tissues.

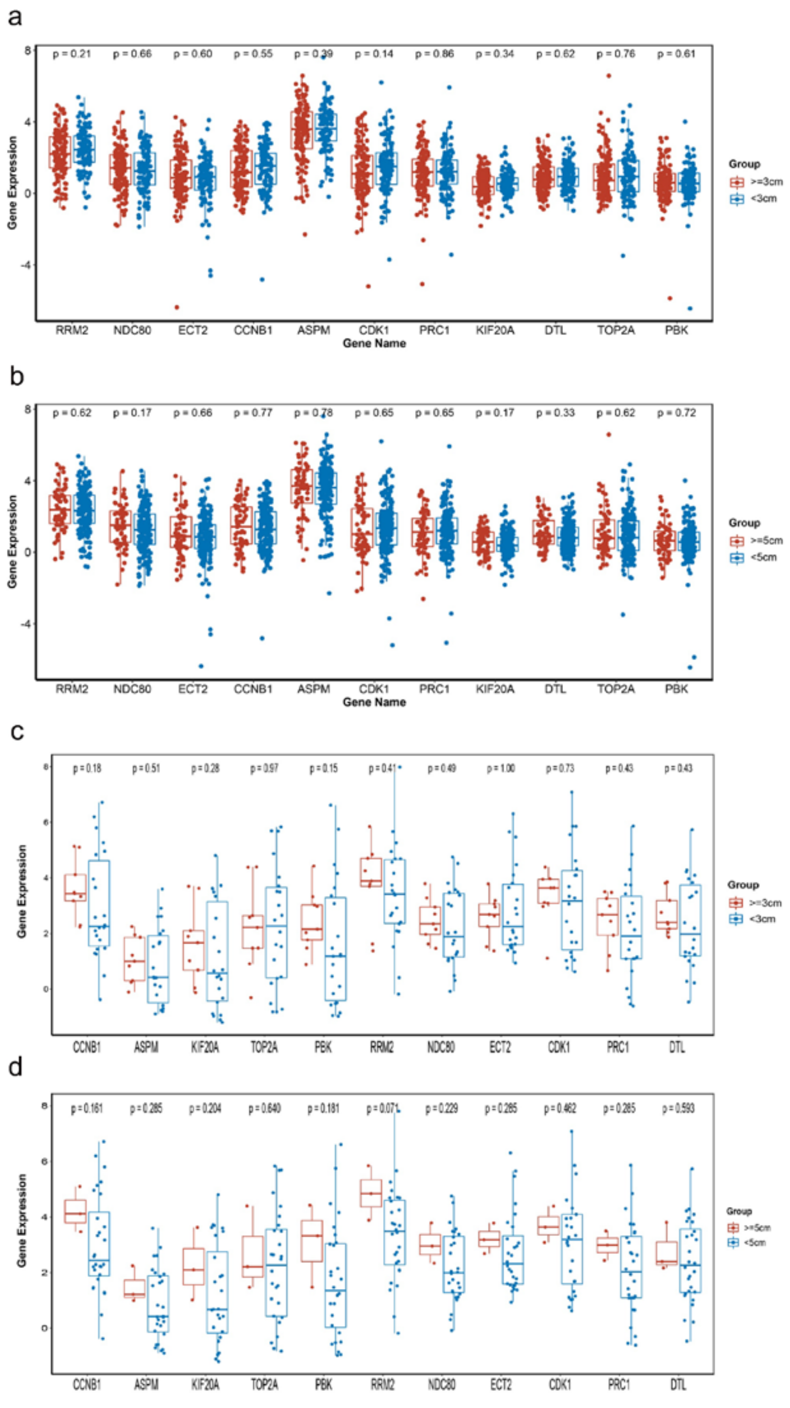

Supplementary Figure S2. Different expression levels of key genes between various tumor size of hepatocellular carcinoma (HCC) patients. (a-b) key genes’ expression in the different tumor size based on Jia Liver data set. (c-d) key genes’ expression in the different tumor size based on Wurmbach Liver data set.

Gene	GSE112790		GSE107170-HBV		GSE107170-HCV		GSE84044	
	logFC	*P* Value	logFC	*P* Value	logFC	*P* Value	logFC	*P* Value
SPINK1	4.58	4.94E-07	4.09	2.31E-22	3.98	1.82E-09	1.02	8.34E-03
GPC3	4.47	7.12E-10	3.61	9.34E-31	3.54	2.44E-19	3.14	4.45E-18
ASPM	3.99	3.77E-19	3.03	1.31E-48	2.06	9.38E-13	1.08	2.58E-04
COL15A1	3.68	2.41E-15	2.06	2.13E-20	1.44	3.82E-05	1.24	6.14E-07
RRM2	3.46	1.07E-17	2.39	8.13E-33	1.12	5.67E-05	1.46	5.65E-05
CDK1	3.42	2.04E-19	2.75	8.96E-41	1.61	7.50E-10	1.14	2.01E-05
DTL	3.35	1.66E-18	2.36	5.03E-30	1.41	5.56E-09	1.26	6.19E-07
TOP2A	3.22	9.94E-15	3.73	1.14E-48	2.06	9.00E-13	1.36	3.01E-06
SPP1	3.10	2.98E-05	3.13	3.84E-16	-1.62	4.49E-03	2.38	1.00E-07
CCNB1	3.06	7.79E-16	2.62	1.65E-41	1.47	8.77E-08	1.04	1.70E-05
NDC80	3.00	6.95E-16	2.86	1.29E-45	1.32	6.55E-11	1.20	9.21E-07
PBK	2.95	1.17E-12	2.60	3.69E-39	1.47	1.71E-09	1.29	5.22E-05
PRC1	2.91	2.35E-16	2.55	4.50E-37	1.52	5.27E-12	1.04	5.63E-05
ECT2	2.67	8.47E-16	2.53	2.12E-31	1.12	1.71E-06	1.09	3.34E-06
KIF20A	2.19	9.96E-11	2.49	9.29E-40	1.55	4.06E-12	1.01	3.92E-05
LEF1	1.95	2.97E-07	2.39	1.17E-20	1.27	5.15E-07	1.04	3.49E-09
ERICH5	1.92	9.22E-04	-1.57	2.51E-07	-1.38	5.99E-03	1.36	3.96E-08
LYZ	1.45	5.13E-04	1.77	7.05E-11	1.30	1.92E-04	1.47	2.75E-10
DKK3	1.16	1.15E-03	2.12	8.00E-12	-1.22	2.54E-04	2.03	4.02E-15
CHST4	-1.00	1.24E-06	-2.01	4.83E-30	-2.79	7.50E-28	1.80	8.34E-14

CCL2	-1.07	3.56E-03	-1.20	2.64E-08	-1.35	6.15E-08	1.46	2.54E-08
FREM2	-1.08	7.34E-04	-3.41	7.40E-40	-3.66	5.24E-39	1.37	9.82E-04
THBS1	-1.19	1.05E-02	-3.23	4.82E-29	-2.76	1.31E-15	1.25	1.02E-07
MYC	-1.24	1.13E-03	-2.09	5.58E-19	-1.94	1.29E-11	1.11	8.49E-09
LOC100505985	-1.27	4.35E-03	-1.81	1.16E-11	-1.00	2.37E-04	-1.23	2.05E-06
FHL2	-1.29	1.53E-04	-1.03	7.33E-06	-1.12	3.31E-05	1.24	6.17E-07
LOC100507389	-1.36	2.91E-02	-1.27	3.25E-05	2.08	7.66E-07	-1.11	2.39E-06
JCHAIN	-1.82	8.09E-04	-3.95	1.10E-22	-2.74	7.62E-11	1.26	5.08E-05
CCN1	-1.86	5.31E-07	-2.24	2.41E-28	-1.50	2.69E-09	1.08	4.45E-05
EGR1	-1.99	4.82E-06	-2.86	1.18E-34	-1.71	4.90E-12	1.03	4.05E-02
HBB	-2.13	1.94E-08	-2.04	1.31E-17	-1.21	7.65E-04	-1.03	4.41E-03
PDGFRA	-2.18	1.97E-05	-2.99	1.73E-18	-2.75	6.32E-11	1.38	7.44E-12
SRPX	-2.32	1.61E-08	-2.97	9.93E-25	-3.12	2.23E-18	1.21	9.06E-11
C7	-2.39	2.23E-05	-4.18	3.44E-25	-2.88	3.85E-12	1.67	8.57E-12
BBOX1	-2.75	4.43E-06	-2.46	4.18E-18	-1.93	7.68E-08	-1.42	3.76E-10
KBTBD11	-2.93	8.43E-22	-1.93	5.19E-31	-1.21	3.93E-07	-1.36	3.46E-10
GNMT	-3.21	1.16E-08	-3.30	1.23E-31	-1.45	4.76E-06	-1.51	2.71E-08
CYP26A1	-3.30	2.40E-21	-3.25	4.37E-39	-1.63	1.51E-15	-1.28	3.54E-03
TTC36	-3.62	1.63E-16	-3.09	1.91E-51	-1.81	5.88E-09	-1.16	1.78E-10
LPA	-3.67	1.08E-18	-2.90	2.83E-28	-1.49	2.08E-07	-1.70	2.40E-09
CLEC4M	-3.75	8.04E-50	-3.23	3.43E-46	-1.57	8.88E-15	-1.08	6.24E-09

AVPR1A	-3.83	2.66E-10	-2.77	1.22E-23	-1.54	7.72E-05	-1.46	8.38E-05
CYP2C19	-4.02	1.16E-50	-1.93	1.21E-27	-1.24	4.76E-12	-2.28	1.07E-07
KCNN2	-4.42	3.41E-32	-3.19	4.35E-33	-1.36	1.87E-04	-2.56	1.33E-12
MT1M	-5.09	5.80E-15	-3.69	4.20E-36	-3.31	8.73E-21	-1.57	1.18E-06
LINC01093	-5.41	1.39E-29	-4.64	1.85E-60	-2.52	5.77E-14	-1.57	4.61E-09

Supplementary Table S1. The co-expressed differentially expressed genes from candidate datasets. HBV, hepatitis B virus, HCV, hepatitis C virus, FC, fold change.
